# Systemic antibiotic prophylaxis does not affect infectious complications in pediatric burn injury: A meta-analysis

**DOI:** 10.1371/journal.pone.0223063

**Published:** 2019-09-25

**Authors:** Alexandra Csenkey, Gergo Jozsa, Noemi Gede, Eszter Pakai, Benedek Tinusz, Zoltan Rumbus, Anita Lukacs, Zoltan Gyongyi, Peter Hamar, Robert Sepp, Andrej A. Romanovsky, Peter Hegyi, Peter Vajda, Andras Garami

**Affiliations:** 1 Department of Thermophysiology, Institute for Translational Medicine, Medical School, University of Pecs, Pecs, Hungary; 2 Department of Paediatrics, Surgical Division, University of Pecs, Pecs, Hungary; 3 Institute for Translational Medicine, Medical School, University of Pecs, Pecs, Hungary; 4 Department of Public Health, Faculty of Medicine, University of Szeged, Szeged, Hungary; 5 Department of Public Health Medicine, Medical School, University of Pecs, Pecs, Hungary; 6 Second Department of Internal Medicine and Cardiology Centre, University Szeged, Szeged, Hungary; 7 Thermoregulation and Systemic Inflammation Laboratory (FeverLab), Trauma Research, St. Joseph's Hospital and Medical Center, Phoenix, Arizona, United States of America; Universidade Federal de Minas Gerais, BRAZIL

## Abstract

In pediatric burns the use of systemic antibiotic prophylaxis is a standard procedure in some burn centers, though its beneficial effect on the infectious complications is debated. The present meta-analysis aimed at determining whether systemic antibiotic prophylaxis prevents infectious complications in pediatric patients with burn injuries. We searched the PubMed, EMBASE, and Cochrane Library databases from inception to August 2019. We included 6 studies, in which event rates of infectious complications were reported in children with burn injuries receiving or not receiving systemic antibiotic prophylaxis. We found that the overall odds ratio (OR) of developing an infection (including local and systemic) was not different between the groups (OR = 1.35; 95% CI, 0.44, 4.18). The chances for systemic infectious complications alone were also not different between antibiotic-treated and non-treated patients (OR = 0.74; 95% CI, 0.38, 1.45). Based on the age, affected total body surface area, and country income level, we did not find any subgroup that benefited from the prophylaxis. Our findings provide quantitative evidence for the inefficacy of systemic antibiotic prophylaxis in preventing infections in pediatric burns. To validate our conclusion, multinational, randomized trials in a diverse population of children with burn injuries are warranted.

## Introduction

Burn injuries in children constitute a major challenge for health care. The incidence and mortality rates of burns show a declining trend worldwide, mainly due to the decreased rates in highly developed countries [1], but several reports indicate an increasing incidence rate of burns in children in well-developed countries like Finland [2], the Netherlands [3], and the Czech Republic [4]. Children accounted for nearly 50% of the population with severe burn injuries in an analysis of studies from 22 European countries, which included data from more than 186,500 patients [5]. The majority of childhood burns occurred in children younger than 5 years of age [5]. In the US, burns were the third leading cause of unintentional injury and death for 1 to 9 year-old children in 2006 [6]. Of 1,559 injured children in low income countries in 2007, burns were also the third most frequent (13%) cause of injuries and had the highest (79%) admission rate among all types of unintentional injuries [7]. According to a recent global estimate, the overall child burn mortality is 2.5 per 100,000, and it is negatively correlated with the economic level of the country, being as high as 9.5 per 100,000 in low income countries such as Mongolia, Rwanda, and Togo [8]. The highest fire-related death rates occur in children younger than four years of age [6].

Burns can be caused by extreme heat (e.g., hot surfaces, fluids, and flame), chemicals, electricity, friction or radiation. Scald burns are the most frequent type of thermal injuries in children under the age of 5 years [5, 6], while between 5 and 16 years of age flame burns are most common [6]. The severity of the burn injury is influenced by several factors, including the nature and duration of the exposure, age and premorbid health and wealth conditions of the child, as well as regional and socioeconomic factors [6, 9]. Burns are classified based on the extent of the damage to the skin layers (depth of burns) and the size of affected skin area, assessed as percentage of total body surface area (TBSA) [10].

Infections, including wound, respiratory, and urinary tract infections, as well as those associated with sepsis, are among the most common complications of burns [11]. In pediatric patients, sepsis is a leading cause of mortality after burn injury, accounting for up to 54% of deaths [12, 13]. Burn wound infections and subsequently sepsis can occur in patients with partial-thickness or full-thickness burn injuries [11]; deeper burns present higher risk for infections [11, 14]. Despite these alarming data, there are no firm rules or guidelines for prophylactic, systemic antibiotic administration in pediatric patients with burn injury. It is recommended that systemic antibiotic administration should be reserved for cases with clear evidence of infection [10], but about 60% of the burn centers in the UK did not have a formal policy on the use of antibiotics, and there was no consensus on antibiotic prophylaxis, according to a study published in 1995 [15]. A more current survey revealed that standard operating procedures were implemented in less than half of UK burn units [16], and a recent study showed notable variations in guideline use for diagnosing and managing infections in pediatric burns [17]. Inappropriate use of antibiotics in burn injuries can increase the chance for complications [18] and result in antibiotic resistance [19], hence it can raise the cost of healthcare to both patients and the community [20]. The present meta-analysis of published clinical trials aims at determining whether systemic antibiotic prophylaxis improves the outcome of pediatric burn injuries. Similar analyses were performed in adult burn patients [21, 22] and helped to form guidelines [23].

## Materials and methods

### Search strategy

The meta-analysis was conducted as described in our recent studies [24, 25]. In brief, we followed the guidelines of the Preferred Reporting Items for Systematic Reviews and Meta-Analysis Protocols [26] (Table A in S1 File). The question of our analysis was formulated with the Participants, Intervention, Comparison, Outcome (PICO) model: in children with burn injury, we aimed to assess the effect of systemic antibiotic prophylaxis on infectious complications. This meta-analysis has been registered with PROSPERO International Prospective Register of Systematic Reviews (registration number: CRD42018102498).

We searched the PubMed, EMBASE, and Cochrane Library databases for relevant articles from inception to August 2019 with the following query: “(antibiotic* OR antimicrobial*) AND (prophylaxis OR prophylactic) AND (burn* OR scald OR flame) AND (pediatric* OR child*)”. Search results were filtered for human studies. The search was conducted separately by two authors (AC, GJ), who also assessed study eligibility and extracted data from the selected studies independently. Disagreements were resolved by consensus, if needed, with the help of a third party (AG). As a specific example for the search, in the EMBASE database, which identified the highest number of articles, the term “(antibiotic* OR antimicrobial*) AND (prophylaxis OR prophylactic) AND (burn* OR scald OR flame) AND (pediatric* OR child*)” was entered and retrieved 230 records, which decreased to 213 studies after the “humans” filter was selected.

### Study selection and data extraction

After screening of the titles and abstracts of the publications identified with the literature search, the full texts of potentially eligible articles were obtained. We included studies which compared event rates of systemic and local complications of burns between children receiving and not receiving systemic antibiotic prophylaxis. Antibiotic prophylaxis was defined as systemic antibacterial drug administration to patients without confirmed infection and systemic inflammatory signs. Wound infection was considered as local complication, while systemic complications included sepsis and suspected toxic shock syndrome.

From the included studies, we extracted the country of origin, characteristics of the patient population (sample size, age, TBSA), and complication events in the different treatment groups (i.e., with or without systemic antibiotic prophylaxis) of children with burn injuries.

To evaluate the quality of the studies included in the meta-analysis, two independent reviewers (AC and BT) assessed the bias of a randomized controlled trial according to the Cochrane Risk of Bias Tool for Randomized Controlled studies [27] (Table B in S1 File), while the quality of other study types was assessed by using the Newcastle-Ottawa Scale [28] (Table C in S1 File).

### Statistical analysis

The statistical analysis was performed according to the standard methods of meta-analysis. Patients were grouped as either treated with systemic antibiotic prophylaxis or not. Pooled odds ratio (OR) with 95% confidence intervals (CI) for infectious complications in pediatric patients with burn injuries were calculated for the dichotomous outcomes. In all forest plots, we applied the random-effect model with DerSimonian-Laird estimation. The OR was calculated by dividing the ratio of events to no events in the antibiotic-treated group with the same ratio in the group without systemic antibiotic prophylaxis. Statistical heterogeneity was determined by the *I*^*2*^ statistical test (*P*<0.1 indicating significant heterogeneity), while publication bias was assessed by the visual inspection of funnel plots (Figs A and B in S1 File), as described elsewhere [24, 25]. Heterogeneity in clinical outcomes was explored by creating different subgroups (age, income, TBSA, type of complication). Sensitivity analysis (i.e., iteratively removing one study from the analyses and recalculating OR to investigate the impact of each individual study on the summary estimate) showed no difference in the final pooled results. The analyses were performed using the Stata 11 SE software (StataCorp LLC, College Station, TX, USA).

## Results

### Study selection

**Fig 1:**
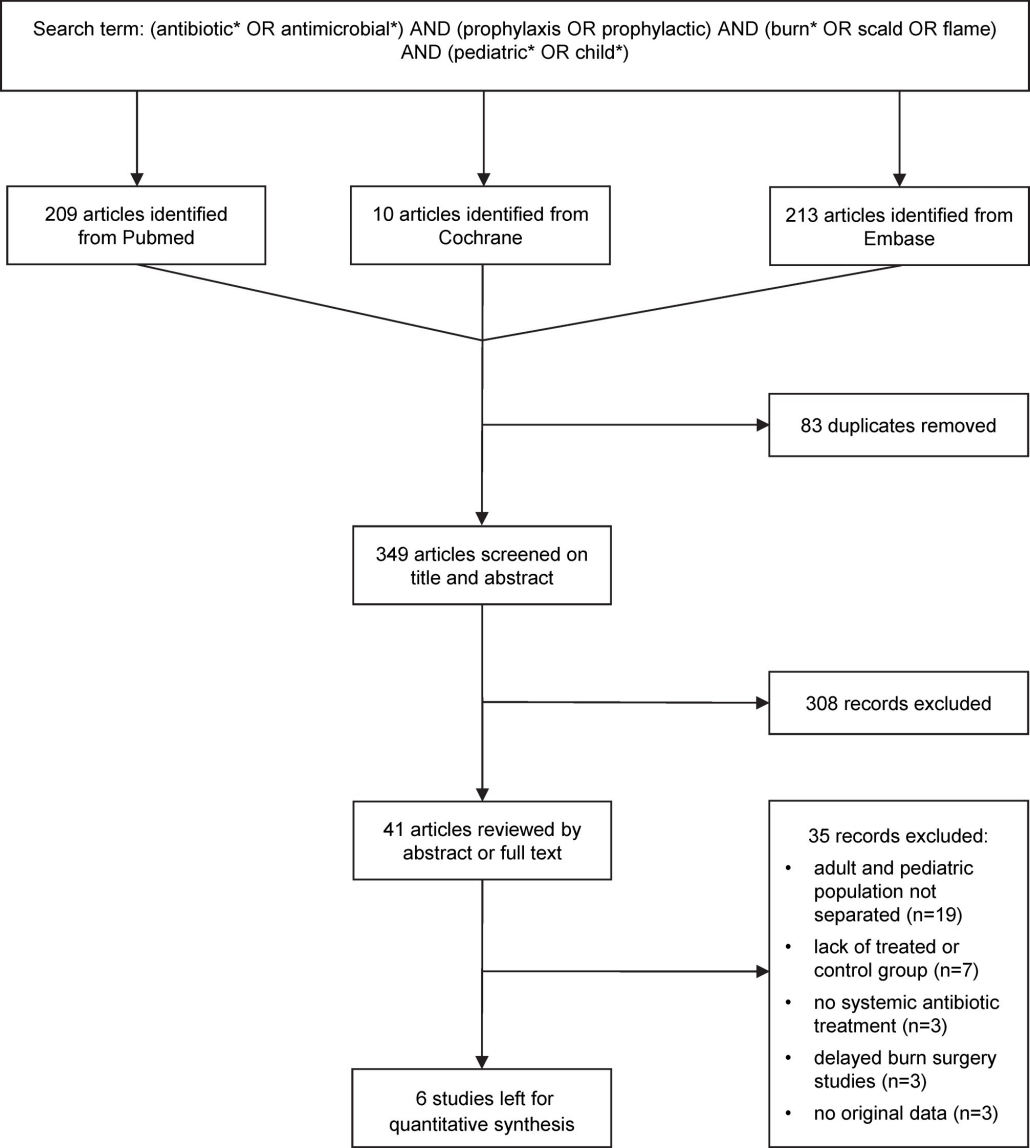
Flow chart of study selection and inclusion.

Fig 1 presents the flow chart of the study selection. Until August 2019 the electronic literature search identified altogether 432 human studies from the PubMed, EMBASE, and Cochrane Library databases. After removing duplicates, 349 articles remained, which were screened on title and abstract for inclusion criteria. Full texts of 41 articles were reviewed and, in the end, 6 publications were found eligible for statistical analysis [18, 29–33], which included data from a total of 1,735 patients. The descriptive characteristics of these studies are shown in Table D in S1 File.

### Effects of systemic antibiotic prophylaxis on local and systemic infectious complications in children with burn injuries

**Fig 2:**
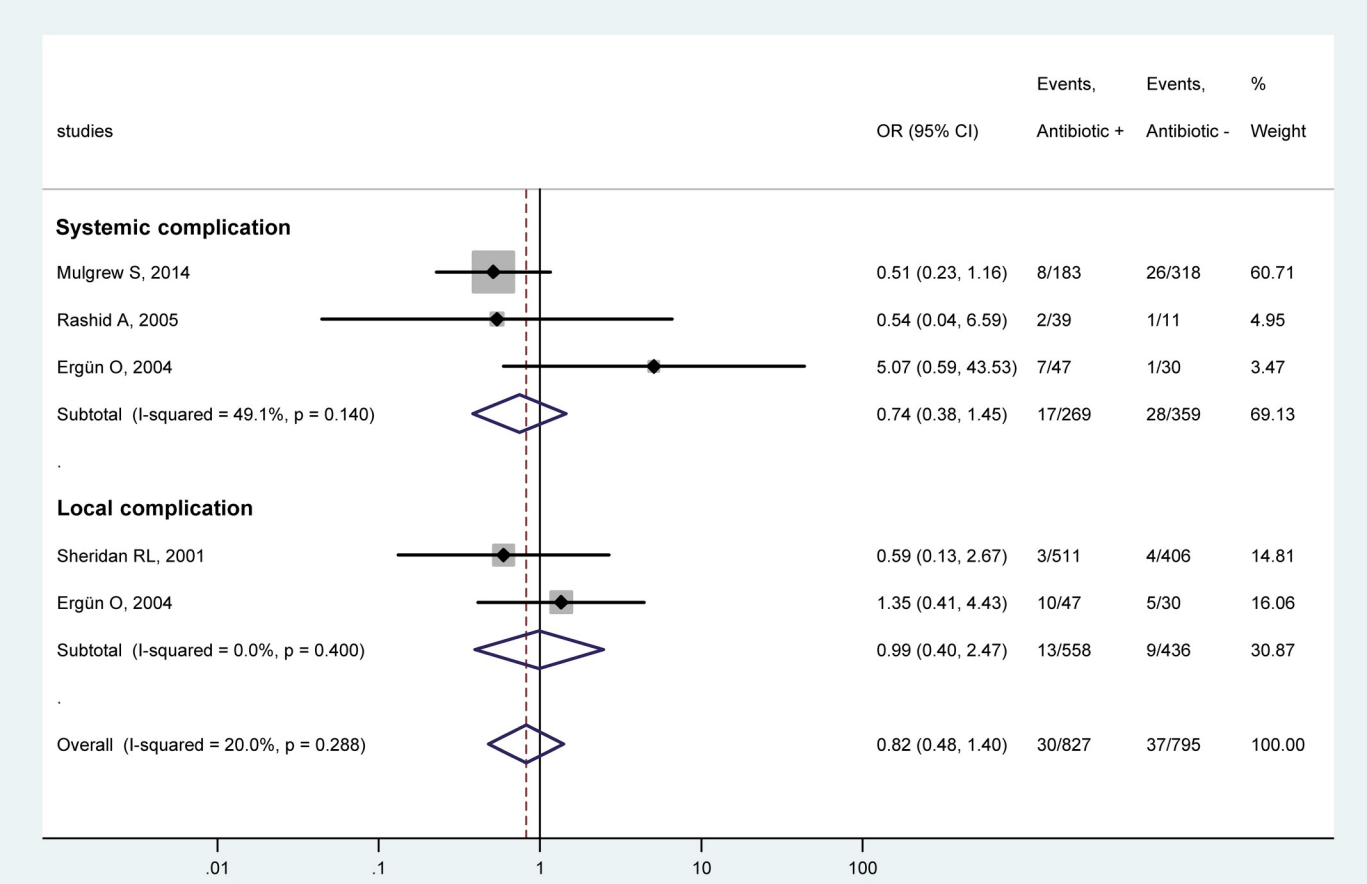
Forest plot of the odds ratios (ORs) for systemic and local subgroups of infectious complications in pediatric patients with burn injuries who received systemic antibiotic prophylaxis compared to those who did not. Here, and in Figs 3–5, black circles represent the OR for each study, while the left and right horizontal arms of the circles indicate the corresponding 95% confidence intervals (CI) for the OR. The size of the gray box is proportional to the sample size of the study; bigger box represents larger sample size, thus bigger relative weight of the study, and vice versa. The diamonds represent the average OR calculated from the ORs of the individual studies in a subgroup (top and middle) and in all studies (bottom). The left and right vertices of the diamonds represent the 95% CI of the average ORs. The vertical dashed line is determined by the low and top vertices of the bottom diamond and indicates the value of the average OR of all studies in the forest plot. An OR lesser than 1 indicates that the use of systemic antibiotic prophylaxis decreased the chance for infectious complications, whereas an OR higher than 1 indicates an increased chance for infections in the antibiotic-treated children.

First, we analyzed whether systemic antibiotic prophylaxis has an effect on the OR for either local or systemic infectious complications. Studies which separately reported the event rates of local [18, 33] or systemic complications [18, 30, 31] were included in the forest plot (Fig 2). Prophylactic administration of systemic antibiotics did not cause a significant change in the odds for systemic infections (OR = 0.74; 95% CI, 0.38, 1.45). With regards to local complications, the use of antibiotics did not have a significant effect in either of the two included studies, however, their averaged result (OR = 0.99; 95% CI, 0.40, 2.47) should be taken with scrutiny due to the low number of studies in this subgroup. The odds of all infectious complications (i.e., both systemic and local) was also not significantly different between the antibiotic-treated and non-treated groups (OR = 0.82; 95% CI, 0.48, 1.40) (Fig 2).

### Chance for infectious complications in different subgroups of pediatric burn patients treated or not treated with systemic antibiotic prophylaxis

Next, we divided the studies into different subgroups according to the known risk factors of the outcome of burns and data availability. Unlike in the first forest plot (Fig 2), where systemic and local complications were distinguished from each other, in the remaining part of our meta-analysis we considered all (i.e., both local and systemic) complications together as the outcome. Using the combined rate of complications allowed us to include two studies in the analysis, in which the separate event rates of local and systemic complications were not reported [29, 32]. Merging the rates of local and systemic complications looked rational, for we did not find a significant difference in the OR between systemic and local complications (Fig 2).

**Fig 3:**
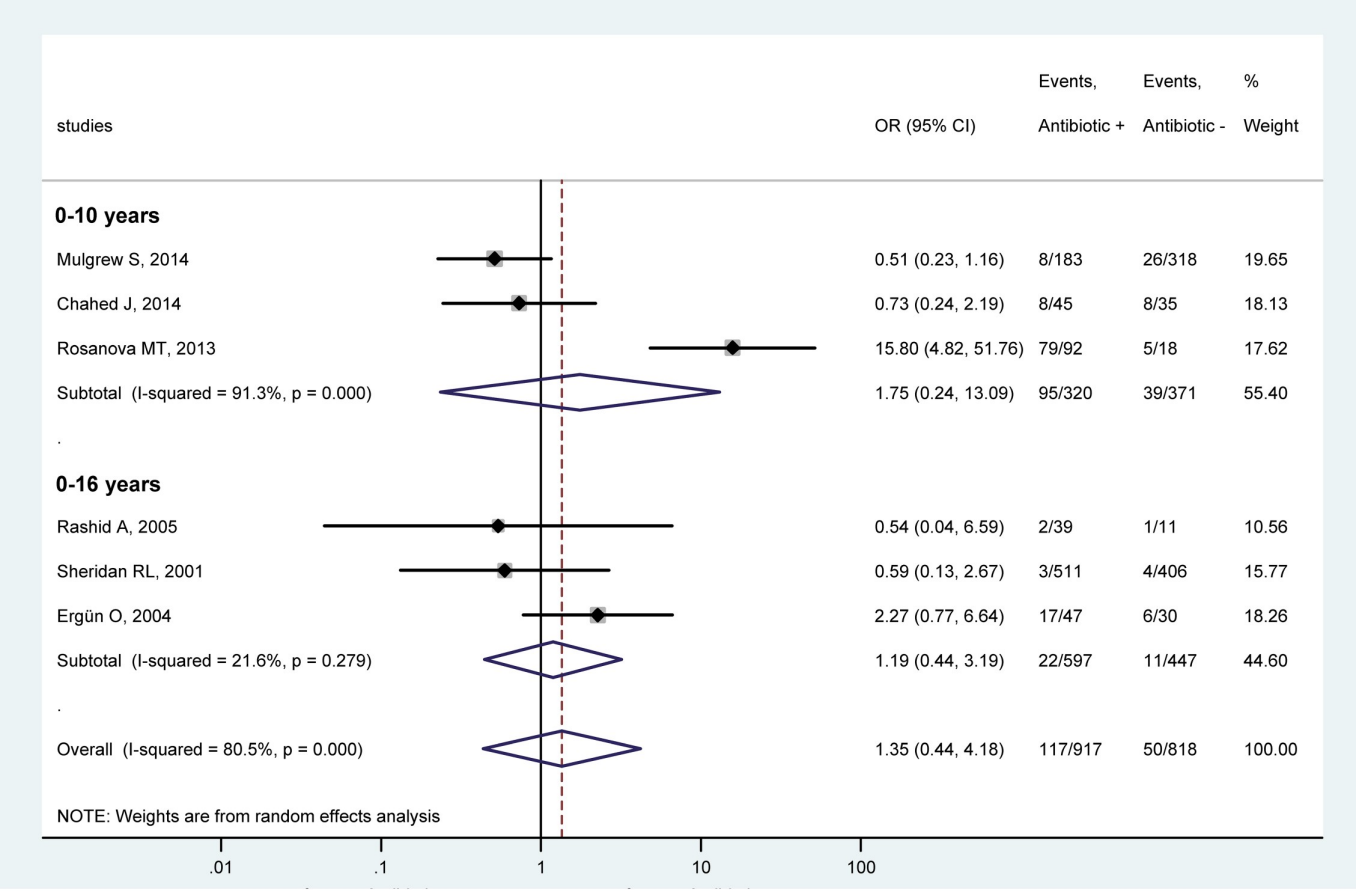
Forest plot of the odds ratios (ORs) for all infectious complications in pediatric patients of 0–10 years (top) and 0–16 years (bottom) with burn injuries who received versus those who did not receive systemic antibiotic prophylaxis.

Based on the age range of the patient populations, the studies were assigned to either of two subgroups: limited to children only, viz., under 10 years of age [29, 30, 32], or also including adolescents up to the age of 16 years [18, 31, 33]. It should be mentioned that if the electronic search was expanded to children and adolescents, the number of eligible studies for quantitative analysis did not increase. Systemic antibiotic prophylaxis did not change the chance for complications in either of the age groups (Fig 3). The OR in the younger (children only) group was 1.75 (95% CI, 0.24, 13.09), while in the older group, which also included adolescents, it was 1.19 (95% CI, 0.44, 3.19). Antibiotic administration did not have any effect on the odds for infections when all 6 studies in the forest plot were combined (OR = 1.35, 95% CI, 0.44, 4.18) (Fig 3).

**Fig 4:**
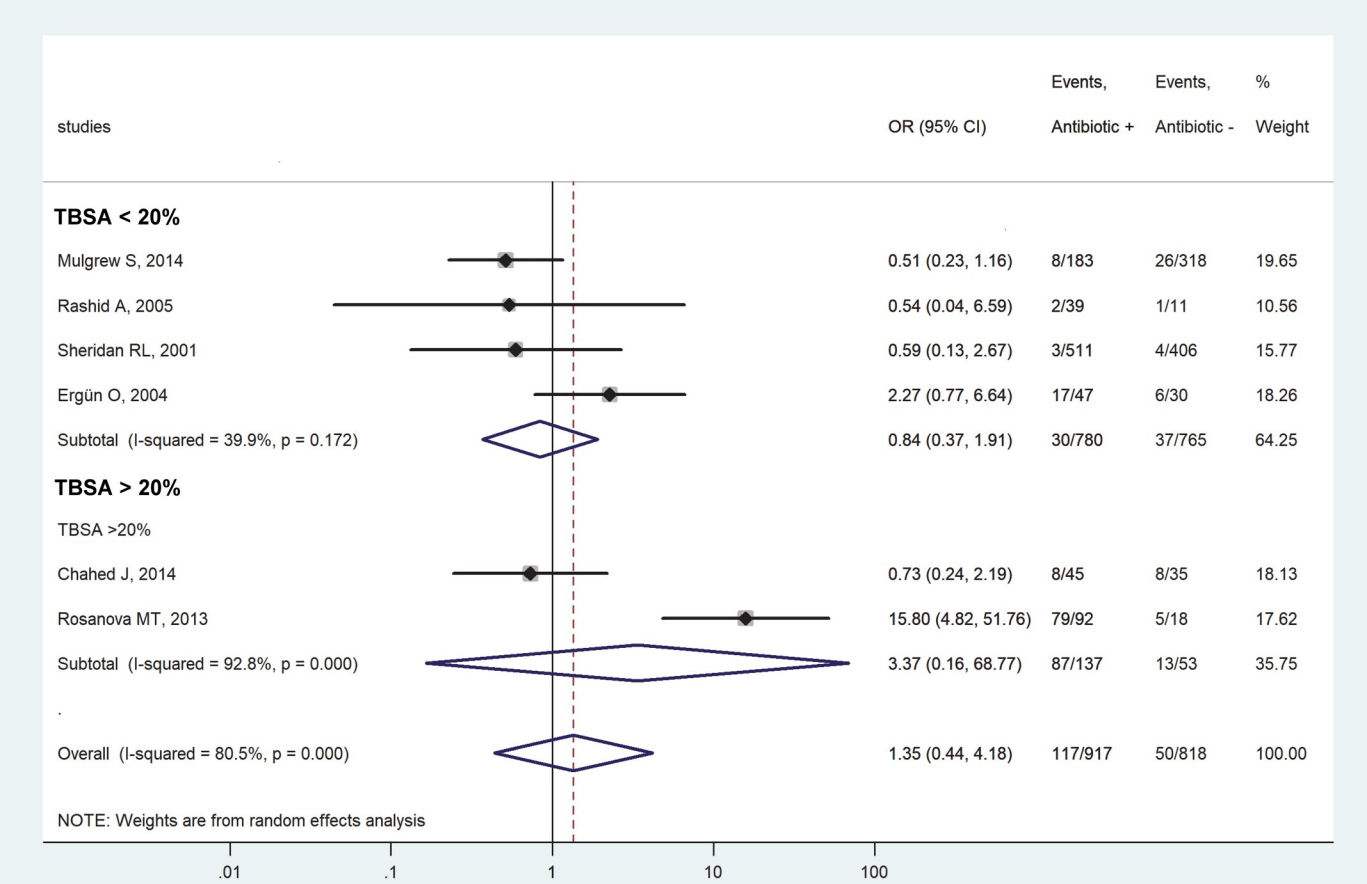
Forest plot of the odds ratios (ORs) for all infectious complications in pediatric patients with burn injuries who received versus those who did not receive systemic antibiotic prophylaxis in subgroups of less than 20% (top) and more than 20% (bottom) mean extent of injury as related to the total body surface area (TBSA).

Based on the mean TBSA affected by the burns, the studies were divided into subgroups of less than 20% [18, 30, 31, 33] and more than 20% of injured TBSA [29, 32]. The reported values of TBSA are included in Table D in S1 File for each study. We did not find a significant effect of systemic antibiotic prophylaxis on the chance of infectious complications in the subgroup consisting of studies with less than 20% affected TBSA (OR = 0.84, 95% CI, 0.37, 1.91) (Fig 4). Though the OR was also not significant in the subgroup with more than 20% of injured TBSA, this group included only 2 studies, which is not sufficient for proper meta-analysis.

**Fig 5:**
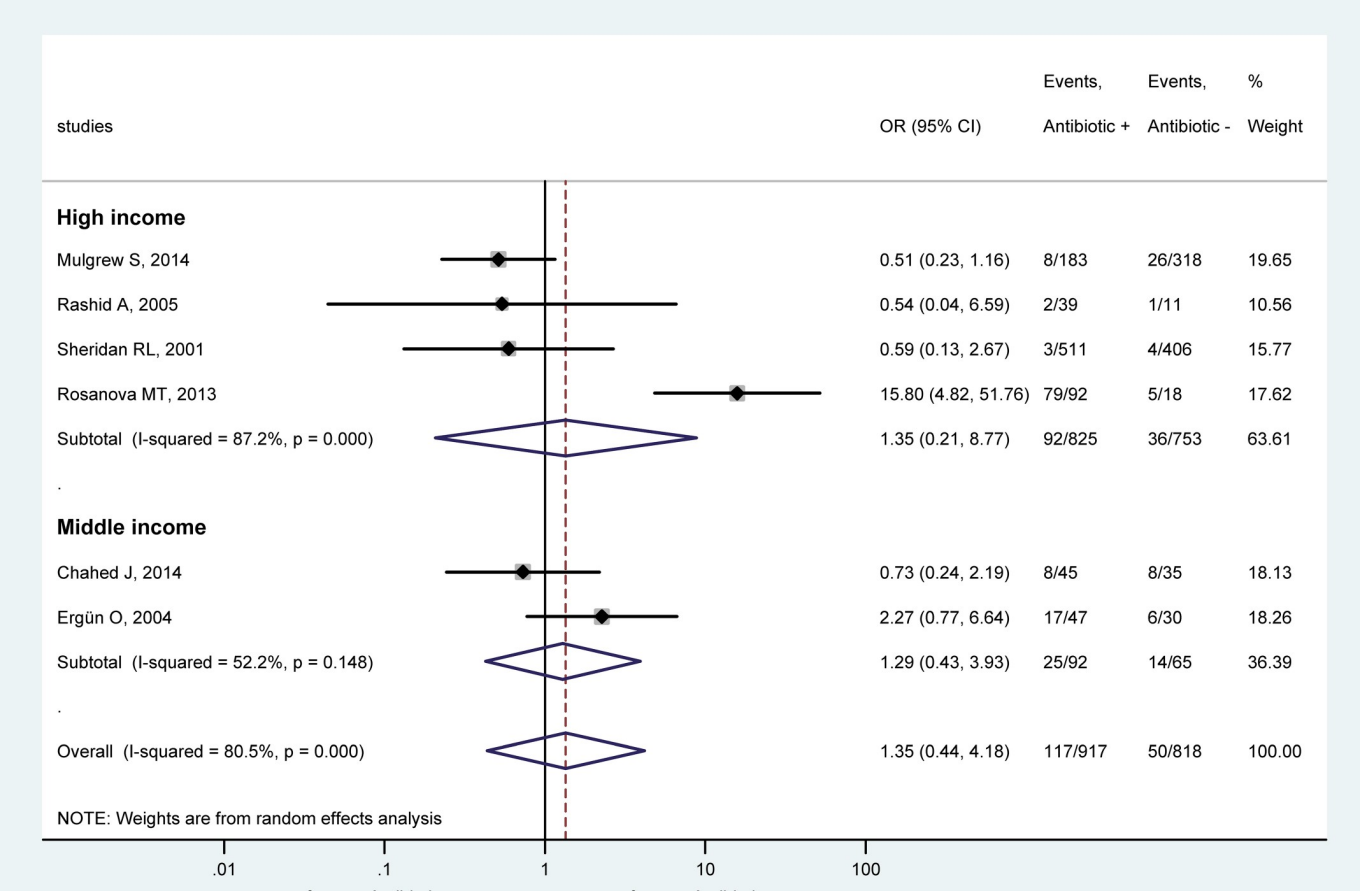
Forest plot of the odds ratios (ORs) for all infectious complications in pediatric patients with burn injuries who received versus those who did not receive systemic antibiotic prophylaxis in country subgroups of high income (top) and middle income (bottom).

Regarding the economic status of the country of the studies, the studies were divided into high-income [30–33] and middle-income subgroups [18, 29] according to classification of the countries in the World Bank Data. Our analysis showed no significant effect of antibiotic prophylaxis on the chance for infections in either of the subgroups. The OR in the high-income subgroup was 1.35 (95% CI, 0.21, 8.77) (Fig 5). The use of antibiotics was also without an effect in either of 2 studies in middle-income countries (Fig 5), but caution is needed regarding their averaged OR due to the low number of studies in this subgroup.

## Discussion

In the present study, we show that systemic antibiotic prophylaxis has no beneficial effects on the risk for infectious complications in pediatric burn injuries. By analyzing data of a total of 1,735 patients, we found that no patient subgroup (based on the age, injured TBSA or country income) benefited (in regards to odds for infectious complications) from receiving prophylactic antibiotic treatment, as compared to burn patients without antibiotic treatment.

Infectious complications are often feared as threats after burn injuries. Superficial burns (traditionally named as first-degree burn), which affect only the epidermis, usually do not require specialized medical care. On the contrary, in deeper burns, which penetrate into the dermis (i.e., partial-thickness, formerly second-degree) or damage the entire dermis and potentially even deeper tissues (i.e., full-thickness, formerly third- and higher-degree burns), the chance of infectious complications is proportionally increasing with the depth of the burn [11]. Deeper burns usually require complex conventional and surgical interventions [10, 34], among them partial-thickness burns are the most common type in children [9, 35, 36].

As part of the primary treatment of deeper burns, systemic antibiotic prophylaxis is occasionally initiated [15, 17], even though there is no clinical evidence for such indication of antimicrobial treatment. In fact, The International Society for Burn Injuries recommends to avoid prophylactic systemic antibiotics in acute burns [23], which guideline is based in part on meta-analyses of data obtained in adult burn patients [21, 22]. In pediatric burns, however, no meta-analysis has been performed, to the best of our knowledge, only a systematic review was published which lacked quantitative statistical analysis [37]. The present work aimed at filling this gap by conducting a meta-analysis of 6 articles [18, 29–33] identified based on an extensive literature search. We showed that systemic antibiotic prophylaxis did not decrease the chance for systemic and all infectious complications. As a matter of fact, when we included the rates of all infectious complications from all 6 eligible studies in our analysis, we found that the overall chance for developing an infection tended to be 35% higher in antibiotic-treated patients (n = 917) than in patients without antibiotic prophylaxis (n = 818), as indicated by the overall OR of 1.35 (95% CI, 0.44, 4.18) (Figs 3–5), although the difference did not reach the level of statistical significance. In accordance, a higher rate of infectious complications was reported in children with burn injuries who received antibiotic prophylaxis in two of the analyzed studies [18, 32]. It was thought to be due to the overgrowth of resistant microorganisms, thereby resulting in infections by opportunistic pathogens in the urinary tract, airways, and middle ear [18]. Antibiotic prophylaxis did not prevent wound infection or potential lethal consequences in the study in 80 pediatric patients with burn injuries conducted by Chahed et al. [29], which was, to our knowledge, the only randomized clinical trial designed to investigate the necessity of systemic antibiotic prophylaxis. Similarly, antibiotic prophylaxis was concluded to be unnecessary in two other studies [30, 33], whereas yet another suggested that prophylactic antibiotics may prevent toxic shock syndrome, based on data obtained from 50 pediatric patients with burn injuries [31]. It has to be noted, however, that in the latter study only 3 patients became septic in the entire study population: 2 (of 39) in the antibiotic-treated group and 1 (of 11) in the group without prophylaxis [31]. Due to the low numbers, these results should be interpreted with caution, as also noted by the authors.

The results of our meta-analysis regarding the lack of efficacy of prophylactic antibiotics on the overall infection rate in pediatric burns are in harmony with the conclusions drawn in the majority of previous human studies [18, 29, 30, 32, 33], a systematic review [37], and recent guidelines [23]. Moreover, by quantitative synthesis of the data reported in the identified articles, our results strengthen the body of evidence for the avoidance of systemic antibiotic prophylaxis in pediatric burns. However, pooling all reported data together and analyzing only the overall infection rate may mask a potentially beneficial effect of antibiotics in a specific subset of pediatric patients. Therefore, we also performed the meta-analysis in different subgroups, which were defined based on known risk factors reported in the identified studies. We found 3 parameters that were reported in sufficient details for subgroup analysis: age, affected TBSA, and country income. We assigned patients to subgroups based on these parameters. Remarkably, there was no statistical difference in the chance of infections between pediatric patients with and without systemic antibiotic prophylaxis in any of the three subgroups. These results suggest that systemic antibiotic prophylaxis should be avoided in pediatric burns independently of the age of pediatric patients, the injured TBSA, or the economic status of the country.

Certain limitations of our study must be also mentioned. Despite the extensive database search, only 6 studies could be included in the final analysis. This was sufficient for quantitative synthesis, but when we divided the studies into subgroups, in some cases only 2 studies per group remained. Although a review of the Cochrane Library revealed that numerous meta-analyses are conducted with two studies [38], firm conclusions should not be drawn from the meta-analysis of such small subgroups. All of the studies included in our meta-analysis were single-center studies, ranging from retrospective [18, 30, 33] to prospective [31, 32] to randomized clinical trials [29]. According to our quality assessment, only 3 studies were considered as good quality [18, 30, 32], while 2 studies as fair [31, 33], and 1 study as poor quality [29]. Based on visual inspection of the funnel plots (Figs A and B in S1 File), some asymmetry may be present, indicating the possible existence of publication bias, but statistical tests could not be performed, because for those at least 10 studies are required according to the Cochrane Handbook [39]. The depths of the burns in the patient populations were not reported in sufficient details to allow for subgroup analysis of the infectious outcome separately in partial- and full-thickness burns. Neither could we extract sufficient data about the latency from the time of the burn injury till initiation and the duration of the systemic antibiotic prophylaxis. Infectious comorbidities (or the lack of such) which had been already present in the children before they suffered burn injury could not be assessed from the studies. Finally, the antibiotics administered to the children varied among the studies. While penicillins were used most commonly for the prophylaxis [18, 29–31, 33], cephalosporins [18, 33] and macrolides [18, 30, 31] were also used in some cases, while, in one study [32], the antibiotic was not identified. All these factors could influence infectious outcomes (whether systemic or local) in pediatric patients with burn injuries, but, due to data unavailability, we could not account for these factors in the present meta-analysis. The mentioned statistical, methodological, and medical differences in study design can explain the considerably high between-study heterogeneity (indicated by an *I*^*2*^ of ~80%), as observed in our analysis (Figs 3–5). To account for the presence of heterogeneity, we used the random-effects model in all forest plots of our meta-analyses. We also performed leave-one-out sensitivity analysis to confirm that our findings were not driven by any single study. However, it is still possible that, despite all of our approaches to reduce methodological errors, the low number, different design and quality, and high heterogeneity of the analyzed studies may have negatively impacted our results.

In conclusion, the present study shows that systemic antibiotic prophylaxis as a routine has no benefits for the prevention of infectious complications in pediatric patients with burn injuries. Our meta-analysis of the data available in literature provides quantitative support to the position of avoiding routine use of the systemic antibiotic prophylaxis in pediatric burns. In addition to the quantitative synthesis of the available data, which to our knowledge, is the first in its field, we point out certain limitations in study design and data reporting, which, however, can also be addressed in the design of future clinical trials. Multinational, randomized controlled trials are warranted to validate our findings and prove unequivocally that routine systemic antibiotic prophylaxis is not indicated in pediatric patients with burn injuries.

## Supporting information

S1 File **Supporting information including Tables A-D and Figures A and B**.
**Table A**. PRISMA 2009 checklist. **Table B**. Risk of bias assessment of a randomized controlled trial included in the meta-analysis using the Cochrane Risk of Bias Tool for Randomized Controlled Trials. **Table C**. Quality assessment of the studies included in the meta-analysis using the Newcastle-Ottawa Scale. **Table D**. Summary of study characteristics for publications included in the meta-analyses. **Figure A**. Funnel plot of the studies that were included in the forest plot of the odds ratios (ORs) for systemic and local subgroups of infectious complications in children with burn injuries who received systemic antibiotic prophylaxis compared to those who did not. **Figure B**. Funnel plot of the studies that were included in the forest plot of the odds ratios (ORs) for all infectious complications in children with burn injuries who received versus those who did not receive systemic antibiotic prophylaxis in the age, TBSA, and country income level subgroups.
(PDF)

## References

[1] Smolle C, Cambiaso-Daniel J, Forbes AA, Wurzer P, Hundeshagen G, Branski LK, et al Recent trends in burn epidemiology worldwide: A systematic review. Burns. 2017;43(2):249–57. 10.1016/j.burns.2016.08.013 . 27600982 PMC5616188

[2] Laitakari E, Koljonen V, Rintala R, Pyorala S, Gissler M. Incidence and risk factors of burn injuries among infants, Finland 1990–2010. J Pediatr Surg. 2015;50(4):608–12. 10.1016/j.jpedsurg.2014.05.034 . 25840072

[3] Vloemans AF, Dokter J, van Baar ME, Nijhuis I, Beerthuizen GI, Nieuwenhuis MK, et al Epidemiology of children admitted to the Dutch burn centres. Changes in referral influence admittance rates in burn centres. Burns. 2011;37(7):1161–7. 10.1016/j.burns.2011.05.001 . 21726947

[4] Celko AM, Grivna M, Danova J, Barss P. Severe childhood burns in the Czech Republic: risk factors and prevention. Bull World Health Organ. 2009;87(5):374–81. 10.2471/BLT.08.059535 . 19551256 PMC2678775

[5] Brusselaers N, Monstrey S, Vogelaers D, Hoste E, Blot S. Severe burn injury in Europe: a systematic review of the incidence, etiology, morbidity, and mortality. Crit Care. 2010;14(5):R188 10.1186/cc9300 . 20958968 PMC3219295

[6] Peck MD. Epidemiology of burns throughout the world. Part I: distribution and risk factors. Burns. 2011;37(7):1087–100. 10.1016/j.burns.2011.06.005 . 21802856

[7] Hyder AA, Sugerman DE, Puvanachandra P, Razzak J, El-Sayed H, Isaza A, et al Global childhood unintentional injury surveillance in four cities in developing countries: a pilot study. Bull World Health Organ. 2009;87(5):345–52. 10.2471/BLT.08.055798 . 19551252 PMC2678776

[8] Sengoelge M, El-Khatib Z, Laflamme L. The global burden of child burn injuries in light of country level economic development and income inequality. Prev Med Rep. 2017;6:115–20. 10.1016/j.pmedr.2017.02.024 . 28316905 PMC5345966

[9] Park JO, Shin SD, Kim J, Song KJ, Peck MD. Association between socioeconomic status and burn injury severity. Burns. 2009;35(4):482–90. 10.1016/j.burns.2008.10.007 . 19216029

[10] Jamshidi R, Sato TT. Initial assessment and management of thermal burn injuries in children. Pediatr Rev. 2013;34(9):395–404. 10.1542/pir.34-9-395 . 24000343

[11] Church D, Elsayed S, Reid O, Winston B, Lindsay R. Burn wound infections. Clin Microbiol Rev. 2006;19(2):403–34. 10.1128/CMR.19.2.403-434.2006 . 16614255 PMC1471990

[12] Geyik MF, Aldemir M, Hosoglu S, Tacyildiz HI. Epidemiology of burn unit infections in children. Am J Infect Control. 2003;31(6):342–6. . 14608300 10.1016/s0196-6553(02)48226-0

[13] Williams FN, Herndon DN, Hawkins HK, Lee JO, Cox RA, Kulp GA, et al The leading causes of death after burn injury in a single pediatric burn center. Crit Care. 2009;13(6):R183 10.1186/cc8170 . 19919684 PMC2811947

[14] Rodgers GL, Mortensen J, Fisher MC, Lo A, Cresswell A, Long SS. Predictors of infectious complications after burn injuries in children. Pediatr Infect Dis J. 2000;19(10):990–5. 10.1097/00006454-200010000-00010 . 11055602

[15] Papini RP, Wilson AP, Steer JA, McGrouther DA, Parkhouse N. Wound management in burn centres in the United Kingdom. Br J Surg. 1995;82(4):505–9. Epub 1995/04/01. 10.1002/bjs.1800820423 . 7613896

[16] Lymperopoulos NS, Jeevan R, Godwin L, Wilkinson D, Shokrollahi K, James MI. The introduction of standard operating procedures to improve burn care in the United Kingdom. J Burn Care Res. 2015;36(5):565–73. Epub 2014/12/17. 10.1097/BCR.0000000000000210 . 25501782

[17] Davies A, Spickett-Jones F, Brock P, Coy K, Young A. Variations in guideline use and practice relating to diagnosis and management of infection in paediatric burns services in England and Wales: A national survey. Burns. 2017;43(1):215–22. 10.1016/j.burns.2016.07.032 . 27597639

[18] Ergun O, Celik A, Ergun G, Ozok G. Prophylactic antibiotic use in pediatric burn units. Eur J Pediatr Surg. 2004;14(6):422–6. 10.1055/s-2004-821065 . 15630646

[19] Soleymanzadeh-Moghadam S, Azimi L, Amani L, Rastegar Lari A, Alinejad F, Rastegar Lari A. Analysis of antibiotic consumption in burn patients. GMS Hyg Infect Control. 2015;10:Doc09 Epub 2015/07/01. 10.3205/dgkh000252 . 26124986 PMC4463254

[20] Thorpe KE, Joski P, Johnston KJ. Antibiotic-resistant infection treatment costs have doubled since 2002, now exceeding $2 billion annually. Health Aff (Millwood). 2018;37(4):662–9. Epub 2018/03/22. 10.1377/hlthaff.2017.1153 . 29561692

[21] Avni T, Levcovich A, Ad-El DD, Leibovici L, Paul M. Prophylactic antibiotics for burns patients: systematic review and meta-analysis. BMJ. 2010;340:c241 10.1136/bmj.c241 . 20156911 PMC2822136

[22] Barajas-Nava LA, Lopez-Alcalde J, Roque i Figuls M, Sola I, Bonfill Cosp X. Antibiotic prophylaxis for preventing burn wound infection. Cochrane Database Syst Rev. 2013;(6):CD008738 10.1002/14651858.CD008738.pub2 . 23740764 PMC11303740

[23] Committee IPG, Steering S, Advisory S. ISBI Practice Guidelines for Burn Care. Burns. 2016;42(5):953–1021. 10.1016/j.burns.2016.05.013 . 27542292

[24] Olah E, Poto L, Hegyi P, Szabo I, Hartmann P, Solymar M, et al Therapeutic whole-body hypothermia reduces death in severe traumatic brain injury if the cooling index is sufficiently high: meta-analyses of the effect of single cooling parameters and their integrated measure. J Neurotrauma. 2018;35(20):2407–17. Epub 2018/04/24. 10.1089/neu.2018.5649 . 29681213

[25] Rumbus Z, Matics R, Hegyi P, Zsiboras C, Szabo I, Illes A, et al Fever is associated with reduced, hypothermia with increased mortality in septic patients: a meta-analysis of clinical trials. PLoS One. 2017;12(1):e0170152 10.1371/journal.pone.0170152 . 28081244 PMC5230786

[26] Moher D, Liberati A, Tetzlaff J, Altman DG, Group P. Preferred reporting items for systematic reviews and meta-analyses: the PRISMA statement. PLoS Med. 2009;6(7):e1000097 10.1371/journal.pmed.1000097 . 19621072 PMC2707599

[27] Higgins JP, Altman DG, Gotzsche PC, Juni P, Moher D, Oxman AD, et al The Cochrane Collaboration's tool for assessing risk of bias in randomised trials. BMJ. 2011;343:d5928 Epub 2011/10/20. 10.1136/bmj.d5928 . 22008217 PMC3196245

[28] Wells GA, Shea B, O’Connell D, Peterson J, Welch V, Losos M, et al The Newcastle-Ottawa Scale (NOS) for assessing the quality of nonrandomised studies in meta-analyses. 2000 [August, 2019]. Available from: http://www.ohri.ca/programs/clinical_epidemiology/oxford.asp.

[29] Chahed J, Ksia A, Selmi W, Hidouri S, Sahnoun L, Krichene I, et al Burns injury in children: is antibiotic prophylaxis recommended? Afr J Paediatr Surg. 2014;11(4):323–5. 10.4103/0189-6725.143141 . 25323182

[30] Mulgrew S, Khoo A, Cartwright R, Reynolds N. Morbidity in pediatric burns, toxic shock syndrome, and antibiotic prophylaxis: a retrospective comparative study. Ann Plast Surg. 2014;72(1):34–7. 10.1097/SAP.0b013e31829be8be . 24056250

[31] Rashid A, Brown AP, Khan K. On the use of prophylactic antibiotics in prevention of toxic shock syndrome. Burns. 2005;31(8):981–5. 10.1016/j.burns.2005.06.017 . 16288963

[32] Rosanova MT, Stamboulian D, Lede R. Infections in burned children: epidemiological analysis and risk factors. Arch Argent Pediatr. 2013;111(4):303–8. 10.1590/S0325-00752013000400008 . 23912287

[33] Sheridan RL, Weber JM, Pasternack MS, Tompkins RG. Antibiotic prophylaxis for group A streptococcal burn wound infection is not necessary. J Trauma. 2001;51(2):352–5. 10.1097/00005373-200108000-00022 . 11493799

[34] Krishnamoorthy V, Ramaiah R, Bhananker SM. Pediatric burn injuries. Int J Crit Illn Inj Sci. 2012;2(3):128–34. 10.4103/2229-5151.100889 . 23181206 PMC3500004

[35] Natterer J, de Buys Roessingh A, Reinberg O, Hohlfeld J. Targeting burn prevention in the paediatric population: a prospective study of children's burns in the Lausanne area. Swiss Med Wkly. 2009;139(37–38):535–9. doi: smw-12605 . 19838870 10.4414/smw.2009.12605

[36] Rawlins JM, Khan AA, Shenton AF, Sharpe DT. Epidemiology and outcome analysis of 208 children with burns attending an emergency department. Pediatr Emerg Care. 2007;23(5):289–93. 10.1097/01.pec.0000248698.42175.2b . 17505269

[37] Lee F, Wong P, Hill F, Burgner D, Taylor R. Evidence behind the WHO guidelines: hospital care for children: what is the role of prophylactic antibiotics in the management of burns? J Trop Pediatr. 2009;55(2):73–7. 10.1093/tropej/fmp017 . 19276147

[38] Turner RM, Davey J, Clarke MJ, Thompson SG, Higgins JP. Predicting the extent of heterogeneity in meta-analysis, using empirical data from the Cochrane Database of Systematic Reviews. Int J Epidemiol. 2012;41(3):818–27. Epub 2012/03/31. 10.1093/ije/dys041 . 22461129 PMC3396310

[39] Higgins JPT, Green S (editors). Cochrane Handbook for Systematic Reviews of Interventions Version 5.1.0 [updated March 2011]. The Cochrane Collaboration, 2011 [August 2019]. Available from: www.handbook.cochrane.org.

