# Supplementary material for: Systemic antibiotic prophylaxis does not affect infectious complications in pediatric burn injury: A meta-analysis
Source: PLoS One. 2019 Sep 25;14(9):e0223063. doi: 10.1371/journal.pone.0223063 (PMC6760783; doi:10.1371/journal.pone.0223063)
Supplement: S1 File — Supporting information including Tables A-D and Figures A and B. Table A. PRISMA 2009 checklist. Table B. Risk of bias assessment of a randomized controlled trial included in the meta-analysis using the Cochrane Risk of Bias Tool for Randomized Controlled Trials. Table C. Quality assessment of the studies included in the meta-analysis using the Newcastle-Ottawa Scale. Table D. Summary of study characteristics for publications included in the meta-analyses. Figure A. Funnel plot of the studies that were included in the forest plot of the odds ratios (ORs) for systemic and local subgroups of infectious complications in children with burn injuries who received systemic antibiotic prophylaxis compared to those who did not. Figure B. Funnel plot of the studies that were included in the forest plot of the odds ratios (ORs) for all infectious complications in children with burn injuries who received versus those who did not receive systemic antibiotic prophylaxis in the age, TBSA, and country income level subgroups. (PDF) [file pone.0223063.s001.pdf]

Supporting information

Table A. PRISMA checklist.

Section/topic	#	Checklist item	Reported on page #
**TITLE**			
Title	1	Identify the report as a systematic review, meta-analysis, or both.	1
**ABSTRACT**			
Structured summary	2	Provide a structured summary including, as applicable: background; objectives; data sources; study eligibility criteria, participants, and interventions; study appraisal and synthesis methods; results; limitations; conclusions and implications of key findings; systematic review registration number.	2
**INTRODUCTION**			
Rationale	3	Describe the rationale for the review in the context of what is already known.	3-4
Objectives	4	Provide an explicit statement of questions being addressed with reference to participants, interventions, comparisons, outcomes, and study design (PICOS).	4
**METHODS**			
Protocol and registration	5	Indicate if a review protocol exists, if and where it can be accessed (e.g., Web address), and, if available, provide registration information including registration number.	5
Eligibility criteria	6	Specify study characteristics (e.g., PICOS, length of follow-up) and report characteristics (e.g., years considered, language, publication status) used as criteria for eligibility, giving rationale.	5
Information sources	7	Describe all information sources (e.g., databases with dates of coverage, contact with study authors to identify additional studies) in the search and date last searched.	5
Search	8	Present full electronic search strategy for at least one database, including any limits used, such that it could be repeated.	5
Study selection	9	State the process for selecting studies (i.e., screening, eligibility, included in systematic review, and, if applicable, included in the meta-analysis).	6
Data collection process	10	Describe method of data extraction from reports (e.g., piloted forms, independently, in duplicate) and any processes for obtaining and confirming data from investigators.	6
Data items	11	List and define all variables for which data were sought (e.g., PICOS, funding sources) and any assumptions and simplifications made.	6
Risk of bias in individual studies	12	Describe methods used for assessing risk of bias of individual studies (including specification of whether this was done at the study or outcome level), and how this information is to be used in any data synthesis.	6
Summary measures	13	State the principal summary measures (e.g., risk ratio, difference in means).	6-7

Synthesis of results	14	Describe the methods of handling data and combining results of studies, if done, including measures of consistency (e.g., I^2) for each meta-analysis.	7
Risk of bias across studies	15	Specify any assessment of risk of bias that may affect the cumulative evidence (e.g., publication bias, selective reporting within studies).	7
Additional analyses	16	Describe methods of additional analyses (e.g., sensitivity or subgroup analyses, meta-regression), if done, indicating which were pre-specified.	7
**RESULTS**			
Study selection	17	Give numbers of studies screened, assessed for eligibility, and included in the review, with reasons for exclusions at each stage, ideally with a flow diagram.	7; Figure 1
Study characteristics	18	For each study, present characteristics for which data were extracted (e.g., study size, PICOS, follow-up period) and provide the citations.	8; Figure 1; Table D
Risk of bias within studies	19	Present data on risk of bias of each study and, if available, any outcome level assessment (see item 12).	6; Tables B and C
Results of individual studies	20	For all outcomes considered (benefits or harms), present, for each study: (a) simple summary data for each intervention group (b) effect estimates and confidence intervals, ideally with a forest plot.	7-11; Figures 2-5
Synthesis of results	21	Present results of each meta-analysis done, including confidence intervals and measures of consistency.	7-11
Risk of bias across studies	22	Present results of any assessment of risk of bias across studies (see Item 15).	6; Figures A and B
Additional analysis	23	Give results of additional analyses, if done (e.g., sensitivity or subgroup analyses, meta-regression [see Item 16]).	9-11
**DISCUSSION**			
Summary of evidence	24	Summarize the main findings including the strength of evidence for each main outcome; consider their relevance to key groups (e.g., healthcare providers, users, and policy makers).	11-14
Limitations	25	Discuss limitations at study and outcome level (e.g., risk of bias), and at review-level (e.g., incomplete retrieval of identified research, reporting bias).	14-15
Conclusions	26	Provide a general interpretation of the results in the context of other evidence, and implications for future research.	15
**FUNDING**			
Funding	27	Describe sources of funding for the systematic review and other support (e.g., supply of data); role of funders for the systematic review.	Submitted separately

Table B. Risk of bias assessment of a randomized controlled trial included in the meta-analysis using the Cochrane Risk of Bias Tool for Randomized Controlled Trials.

Chahed 2014

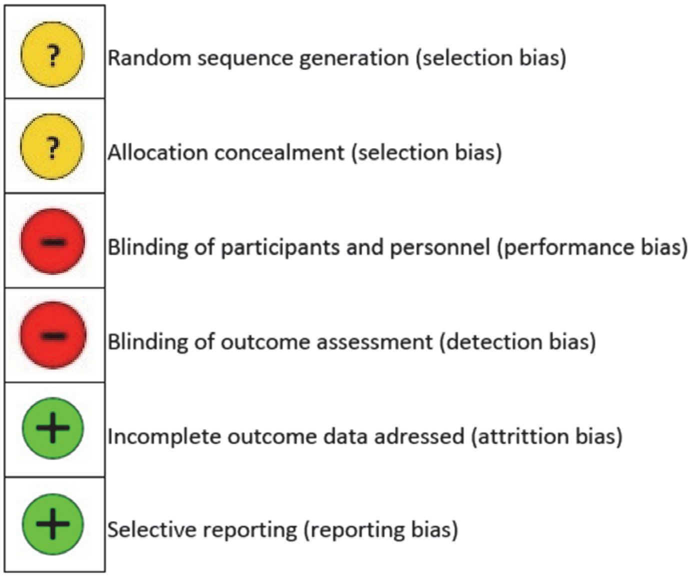

According to the Cochrane Risk of Bias Tool, the study is considered overall as poor quality, for two of the points were determined as "potentially high risk of bias".

+, low risk; -, high risk; ?, unclear risk of bias.

Table C. Quality assessment of the studies included in the meta-analysis using the Newcastle-Ottawa Scale. A score of 7 to 9 indicates a good, a score of 4 to 6 a fair, and a score of 0 to 3 a low methodological quality.

		Representativeness of the exposed cohort	Selection of the non-exposed cohort	Ascertainment of the therapy	Demonstration that outcome of interest was not present at start of study	Comparability of cohorts on the basis of the design or analysis controlled for confounders	Assessment of outcome	Was follow-up long enough for outcomes to occur	Adequacy of follow-up of cohorts		
Ergün O	2003	*	*	*	*	*	*	*		7 stars	Good quality
Sheridan RL	2001	*		*		*	*	*		5 stars	Fair quality
Rosanova MT	2013	*	*	*	*	*	*	*	*	8 stars	Good quality
Mulgrew S	2014	*	*	*	*	*	*	*		7 stars	Good quality
Rashid A	2005	*	*	*		*	*	*		6 stars	Fair quality

Table D. Summary of study characteristics for publications included in the meta-analyses.

First author (year)	Country	Country income level	Antibiotic-treated / non-treated patients (n)	Local / systemic / all infectious complications (n)		Age range (years)	Mean injured TBSA (%)	Depth of burn	Duration of antibiotic treatment (days)
				Antibiotic-treated	Non-treated				
Sheridan (2001)	USA	High	511/406	3/NR/3	4/NR/4	0-16	11-12	Superficial	3
Ergun (2004)	Turkey	Middle	47/30	10/7/17	5/1/6	0-16	10-18	Superficial to full thickness	NR
Rashid (2005)	UK	High	39/11	NR/2/2	NR/1/1	0-12	6	Partial to full thickness	1
Rosanova (2013)	Argentina	High	92/18	NR/NR/79	NR/NR/5	0-2	27*	Superficial to deep	NR
Chahed (2014)	Tunisia	Middle	45/35	NR/NR/8	NR/NR/8	0.8-8	27	Second to third degree	NR
Mulgrew (2014)	UK	High	183/318	NR/8/8	NR/NR/26	0-5	7	NR	5

NR, not reported; TBSA, total body surface area; *median of injured TBSA

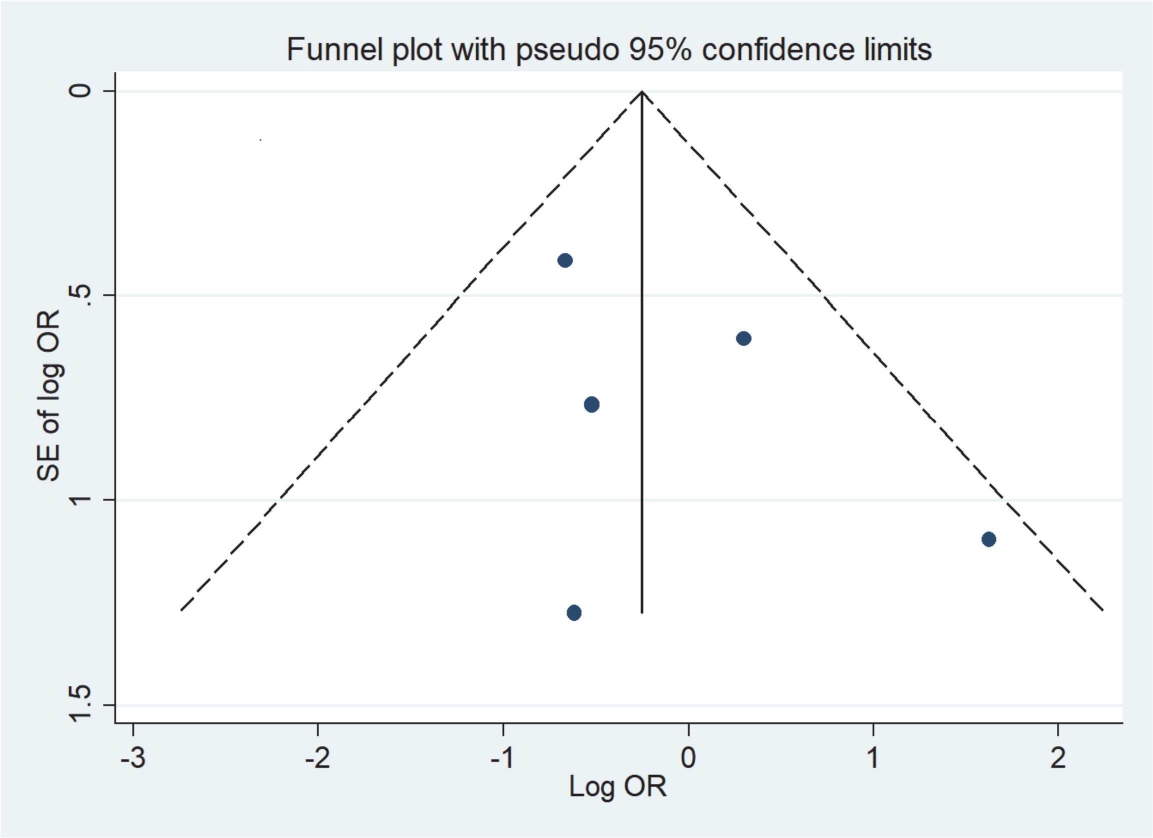

Figure A. Funnel plot of the studies that were included in the forest plot of the odds ratios (ORs) for systemic and local subgroups of infectious complications in children with burn injuries who received systemic antibiotic prophylaxis compared to those who did not.

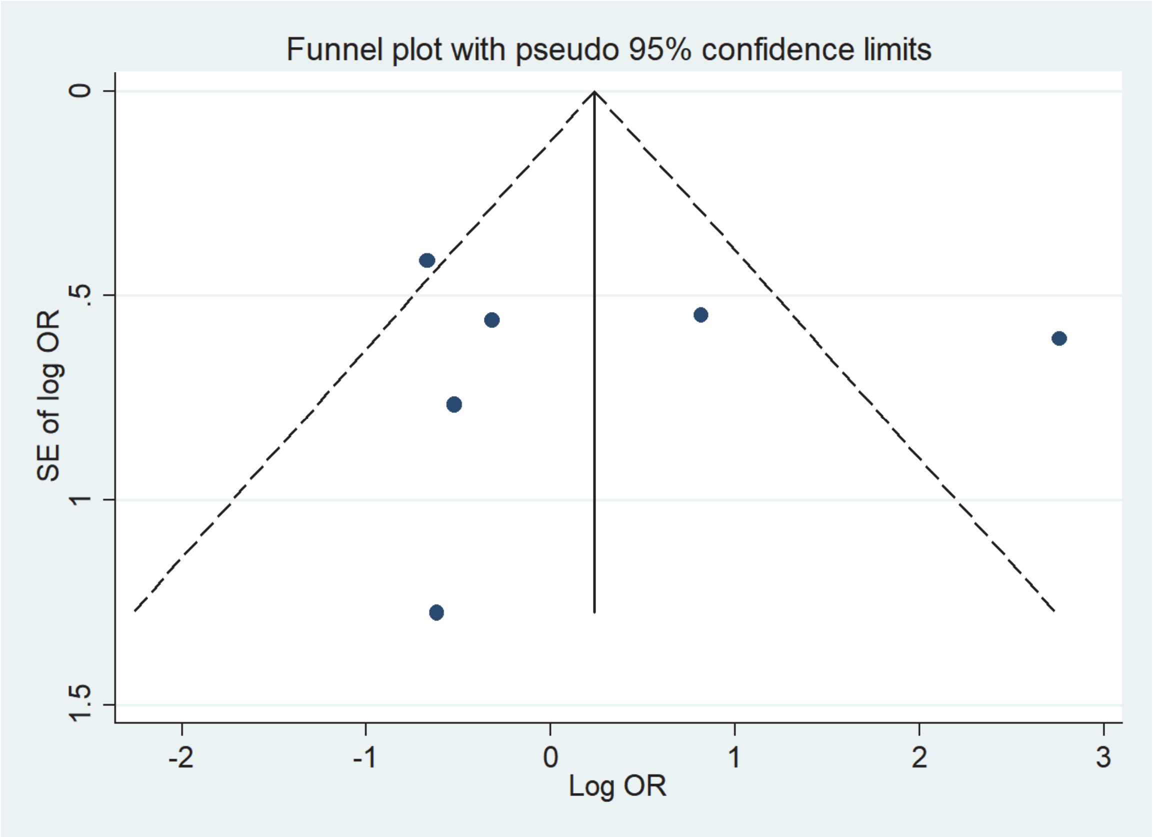

Figure B. Funnel plot of the studies that were included in the forest plot of the odds ratios (ORs) for all infectious complications in children with burn injuries who received versus those who did not receive systemic antibiotic prophylaxis in the age, TBSA, and country income level subgroups.
